# Long-term follow-up of systemic onset juvenile idiopathic arthritis patients treated with Anakinra

**DOI:** 10.1186/1546-0096-9-S1-P158

**Published:** 2011-09-14

**Authors:** Marco Gattorno, Aldo Naselli, Andrea Accogli, Sabrina Chiesa, Roberta Caorsi, Jessica Tebaldi, Antonella Buoncompagni, Nicola Ruperto, Stefania Viola, Paolo Picco, Angelo Ravelli, Alberto Martini

**Affiliations:** 1 2nd Division of Pediatrics, “G. Gaslini” Institute, Genoa, Italy

## Background

Anakinra is effective in a subgroup of SoJIA patients

## Aim

To analyze the long-term follow-up of Anakinra-treated patients

## Methods

34 SoJIA patients (19 M, 15 F) were treated with Anakinra at the staring dose of 1 mg/kg/die. Complete response: no clinical and laboratory activity in absence of other treatments; partial response: active disease despite anakinra; no responders: withdraw of Anakinra due to inefficacy.

## Results

The mean follow-up was 4.02 year (range 1.05 - 6.16). At the last follow-up 13 patients were complete responders, 5 partial responders and 16 non responder.

Among complete responders, 4 patients withdrawn Anakinra without relapses after a mean of 3 years of treatment, 7 are in remission using anakinra only, 2 patients were switched to anti IL-1 monoclonal antibody with a full response. Despite the good control of their disease 11/13 displayed at least one relapse of their disease during the follow-up with a total of 22 relapses (range 1-4 for patient).

Among partial responders two patients were previously considered as complete responders. In 16 non responders patients subsequent treatments were canakinumab (1 pt), tocilizumab (5 patients), or combined immunosuppressive treatment and/or anti-TNF (10 pts). Two non-responders patients died. Responders patients confimed to have an higher number of active joints at baseline (p = 0.006) and higher WBC and neutrhophils count (p = 0.002). Newly enrolled responder patients has a significantly shorter disease duration in respect to non responder patients (p = 0.03).

## Conclusions

Anakinra response still dissect two distinct populations of SoJIa patients

